# Neo-Epitopes—Fragments of Cartilage and Connective Tissue Degradation in Early Rheumatoid Arthritis and Unclassified Arthritis

**DOI:** 10.1371/journal.pone.0149329

**Published:** 2016-03-28

**Authors:** Karen I. Maijer, Natasja Stæhr Gudmann, Morten Asser Karsdal, Daniëlle M. Gerlag, Paul Peter Tak, Anne Christine Bay-Jensen

**Affiliations:** 1 Division of Clinical Immunology and Rheumatology, Academic Medical Center/University of Amsterdam, Amsterdam, the Netherlands; 2 Nordic Bioscience, Herlev, Denmark; 3 University of Southern Denmark, Odense, Denmark; Baylor College of Medicine, UNITED STATES

## Abstract

**Objective:**

Tissue destruction in rheumatoid arthritis (RA) is predominantly mediated by matrix metalloproteinases (MMPs), thereby generating protein fragments. Previous studies have revealed that these fragments include MMP-mediated collagen type I, II, and III degradation, citrullinated and MMP-degraded vimentin and MMP degraded C-reactive protein. We evaluated if biomarkers measuring serum levels of specific sequences of the mentioned fragments would provide further information of diagnostic and/or prognostic processes in early arthritis.

**Methods:**

Ninety-two early arthritis patients (arthritis duration<1 year, DMARD naïve) were enrolled. Patients either fulfilled the ACR/EULAR2010 criteria for RA (n = 60) or had unclassified arthritis (UA) (n = 32). Patients fulfilling the RA criteria after 2 years follow-up were classified into *non-erosive* (n = 25), or *erosive disease* (n = 13). Concentrations of the biomarkers: C1M, C2M, C3M, VICM and CRPM were measured in baseline serum.

**Results:**

C1M, C3M and CRPM were able to discriminate between the UA and RA baseline diagnosis in 92 patients with an AUROC of 0.64 (95%CI 0.517 to 0.762), 0.73 (95%CI 0.622 to 0.838) and 0.68 (95%CI 0.570 to 0.795). C2M showed a potential for discrimination between *non-erosive* and *erosive disease in 38 patients* with an AUROC of 0.75 (95%CI 0.597 to 0.910). All of the applied biomarkers correlated with one or more of the disease activity parameters: DAS28, ESR, CRP, SJC66, TJC68 and/or HAQ.

**Conclusion:**

This is the first study evaluating the applied biomarkers at this early stage of arthritis. C1M, C3M, CRPM might be the best diagnostic marker, whereas high levels of C2M indicated progression of disease at follow-up in early RA patients.

## Introduction

Rheumatoid arthritis (RA) is a systemic autoimmune disease of unknown etiology, characterized by synovial inflammation in multiple joints [1]. Moreover, RA is associated with excessive turnover of connective tissues of the joints, specifically the extracellular matrix (ECM) in cartilage, bone and synovium. Consequently joints become damaged and disabled [2,3].

Throughout the last 25 years several treatment regimens have been developed, but none of them are effective in all patients [4,5]. It is therefore of interest to subclassify patients for further characterization of the pathogenesis of the disease, which may lead to a better understanding of the disease [6,7]. Early detection of joint damage may be identified and characterized by biochemical markers that predict which patients have severe ongoing joint damage and hence are in need of most aggressive treatment [8,9].

The ECM of the cartilage consists mainly of type II collagen, while type I and III collagens are the main proteins of soft tissue surrounding the joint, as the synovium and entheses [10]. Examining the turnover of these and other collagens may aid the understanding of RA pathogenesis. In RA, inflammation leads to excessive remodelling and tissue turnover. Tissue destruction of the ECM in RA is mediated by enzymatic cleavage predominantly by matrix metalloproteinases (MMPs). MMPs have been shown to be highly up-regulated in RA [11,12]. Consequently, a range of protein-degradation products are generated, which results in the exposure of *de novo* sites of these fragmented proteins, referred to as neo-epitopes [13]. Moreover, these protein-degradation products may be specific for the tissue of origin and for the involved enzymes, and may therefore be used as diagnostic and prognostic biomarkers [14].

Such biomarkers include C1M, which is a product of MMP-cleavage of type I collagen and a biomarker of soft tissue destruction [15]. This biomarker has proved its value in RA as it is able to depict fast progressors from slow progressing disease in the phase III tocilizumab trial LITHE [15]. C1M in combination with MMP3 and CRPM were able to predict, which patients had an increased chance of responding to treatment in the LITHE study [16]. CRPM is the MMP-depended degradation product of C-reactive protein (CRP) [17]. Other soluble biomarkers of interest include C2M, C3M, and VICM. C2M is a serum biomarker that measures a MMP-generated neo-epitope of type II collagen, thereby reflecting cartilage degradation [18]. C3M is a biomarker of soft tissue turnover associated with inflammation [19,20], and VICM evaluates citrullinated and MMP-degraded vimentin [21].

Since all of the mentioned biomarkers have proved useful in evaluation and characterization of established RA [15,16] the objective of this study was to evaluate and characterize the tissue turnover of the joints as reflected by C1M, C2M, C3M, VICM, and CRPM in early arthritis patients. Furthermore, we studied whether these biomarkers could provide additional information for the diagnostic and/or prognostic process in the very early phase of inflammatory arthritis, when peripheral blood samples are collected during the patient’s first visit to the rheumatology department.

## Materials and Methods

### Patients

Ninety-two early arthritis patients were enrolled in the prospective early arthritis ‘Synoviomics’ cohort at the Academic Medical Center (AMC) in Amsterdam between April 2004 and January 2013 in this study [22]. At baseline the selected patients either fulfilled the ACR/EULAR 2010 criteria for RA classification (n = 60) [23] or had unclassified arthritis (UA) that did not fulfill classification criteria of established rheumatic disease (n = 33) (phase e according to the EULAR Study Group for Risk Factors for Rheumatoid Arthritis) [24]. All patients enrolled in the study had less than 1 year disease duration, as measured from the first clinical evidence of joint swelling. Patients had active arthritis in at least one joint and were disease-modifying antirheumatic drug (DMARD) naïve. All patients provided written informed consent. The study was performed according to the Declaration of Helsinki and approved by the Medical Ethics Committee of the Academic Medical Center (AMC).

### Study design

At baseline, demographic data were collected and the following clinical and laboratory parameters were obtained: serum levels of C-reactive protein (CRP); erythrocyte sedimentation rate (ESR); 68 tender and 66 swollen joint count (TJC68 and SJC66); Disease Activity Score in 28 joints (DAS28); IgM-RF levels using IgM-RF ELISA (Sanquin, Amsterdam, the Netherlands (upper limit of normal (ULN) 12.5 IU/ml)) until December 2009 and thereafter using IgM-RF ELISA (Hycor Biomedical, Indianapolis, IN (ULN 49 IU/ml)); anti-citrullinated protein antibodies (ACPA) using anti-citrullinated cyclic peptide (CCP)2 ELISA CCPlus (Eurodiagnostica, Nijmegen, the Netherlands (ULN 25 kAU/l)); and radiographs of hands and feet.

Patients were followed for 2 years and those with UA were categorized for diagnostic outcome as having either converted to RA (UA-RA; n = 6) or remained unclassified (UA-UA; n = 23). Three patients were not available for follow-up, and were therefore excluded from the diagnostic outcome analysis. Patients fulfilling the ACR/EULAR 2010 criteria for RA after 2 years follow-up were further classified for prognostic outcome into: *(1) non-erosive disease* (n = 25), or *(2)erosive disease* (n = 13), defined as presence of joint erosions on radiographs of the hands and/or feet [25]. The group of *non-erosive disease* consisted of patients with self-limiting disease (n = 3), and persistent non-erosive disease (n = 22). Self-limiting disease was defined as no arthritis on examination and no use of DMARDs or steroids in the preceding three months. Persistent disease was defined as the presence of arthritis in at least 1 joint and/or DMARDs or steroids use in the preceding three months but who had no evidence of joint erosion. The prognostic outcome data were not available for 28 of the patients and were therefore excluded from the prognostic outcome analysis. Finally, patients were classified as being in remission (DAS < 2.6) or not (DAS ≥2.6) [26].

### Biomarker measurements

Levels of five protein biomarkers (MMP degraded type I collagen [C1M], cartilage degradation [C2M], MMP degraded type III collagen [C3M], citrullinated and degraded vimentin [VICM], and MMP-degraded CRP [CRPM]) were measured in baseline patient serum samples. Measurements were performed manually on blinded samples using competitive enzyme-linked immune sorbent assays (ELISAs) developed and produced by Nordic Bioscience (Herlev, Denmark).

Briefly, for C1M; 96-well streptavidin plates (Roche Diagnostics, Mannheim, Germany) were coated with biotinylated synthetic peptide Biotin-K-GSPGKDGVRG dissolved in assay buffer (50 mM Tris, 1% BSA, 0.1% Tween-20, pH 7.4) adjusted and incubated 30 min at 20°C. 20 μL of peptide calibrator or sample were added to appropriate wells, followed by 100 μL of conjugated monoclonal antibody 4D3-HRP and incubated 1 hour at 20°C. Finally, 100 μL/well tetramethylbenzinidine (TMB) (Kem-En-Tec cat. no. 438OH) was added and the plates were incubated 15 min at 20°C in the dark. When C2M was measured; 4 ng/mL of biotin-KPPGRDGAAG (American peptide, Sunnyvale, CA) was coated onto the streptavidin pre-coated 96-well plates (Roche Diagnostics, Mannheim, Germany) and left for 30 min at 20°C. The calibrators, controls, and undiluted serum samples were added followed by peroxidase-conjugated monoclonal antibody NB44-3C1, and incubated at 4°C for 20 hours. The peroxidase reaction was visualized by 15 min incubation with 3,3′,5,5′-tetramethylbenzidine (TMB, Kem-En-tec, Taestrup, Denmark) at 20°C. For C3M, 96-well streptavidin-coated plates (Roche Diagnostics, Mannheim, Germany) were coated with 0.4 ng/mL of KNGETGPQGP-biotin and left for 30 min at 20°C. Calibrators, controls, and serum samples (diluted 1:1 in incubation buffer) were added, followed by peroxidase-conjugated antibody NB51-G12. The sample—antibody mix was incubated at 20°C for 60 min. TMB was added afterwards and incubated at 20°C and stopped after 15 min. CRPM measurement followed the same procedure as C3M; however, applying a different peroxidase-conjugated antibody (NB94-1A7) and coater (KAFVFPKESDK-biotin). For measurement of serum, VICM samples were prediluted 4 times in incubation buffer. Streptavidin-coated 96-well plates were coated with 100 μL biotin—RLRSSVPGV—citrulline and left for 30 min at 20°C. The calibrators, controls, and prediluted serum samples were added followed by 100 μL of peroxidase-conjugated monoclonal antibody NB212-1C5 and incubated at 4°C for 20 hours. Afterwards sample/calibrator incubation 100 μL of TMB was added and plates were incubated at 20°C for 15 min. All of the mentioned incubation steps included shaking at 300 rpm. After each incubation step the plate was washed five times in washing buffer (20 mM Tris, 50 mM NaCl, pH 7.2). The TMB reaction was stopped by adding 100 μL of stopping solution (0.1M sulfuric acid) and measured at 450 nm with 650 nm as the reference. Calibration curves were plotted using a 4-parametric mathematical fit model.

### Statistical analysis

Categorical data were depicted as number (%) and differences between study groups analyzed using Chi-square test. Not normally distributed variables were depicted as median (interquartile range, IQR). To compare baseline characteristics and biomarker concentration between the different classification groups, the Kruskal-Wallis test was used when more than 2 groups were compared: subsequently the Mann-Whitney U test was used to compare differences between two subgroups. Bivariate correlations of not normally distributed variables were analyzed using Spearman’s rank correlation test. In order to assess the discriminating power of the biomarkers studied we generated ROC curves by using baseline diagnosis (UA or RA), by using diagnostic outcome of the UA patients only (UA-RA or UA-UA), by mean of prognostic outcome (non-erosive or erosive disease) and by classifying patients into remission or not as outcomes. To examine the relationship between the biomarkers and baseline diagnosis, diagnostic outcome, and prognostic outcome, we performed binary logistic regression. All statistical analyses were performed by using SPSS v20.0 software (IBM Corp., Armonk, NY) and MedCalc version 14.8.1 (MedCalc Software bvba, Ostend, Belgium). A p-value of <0.05 was considered statistically significant. Bonferroni correction was applied to correct for multiple comparisons.

## Results

### Early arthritis patients

Baseline characteristics of the early arthritis patients are shown in Table 1.

**Table 1:** Baseline characteristics of early arthritis patients.

	Rheumatoid arthritis	Unclassified arthritis	p Value
Characteristic	N = 60	N = 32	
Sex, female (n (%))	42 (70)	17 (53)	0.11
Age, years (mean (SD))	53 (40–61)	46 (34–59)	0.097
IgM-RF positive (n (%))	35 (58)	1 (3)	**<0.001**
ACPA positive (n (%))	44 (73)	3 (9)	**<0.001**
IgM-RF and ACPA bothpositive (n (%))	30 (50)	0	**<0.001**
ESR, mm/hr (median (IQR))	17 (7–37)	9 (5–22)	0.079
CRP, mg/L (median (IQR))	6.4 (1.6–16.1)	3.0 (1.2–7.9)	0.12
DAS28 (median (IQR))	4.7 (3.3–6.1)	3.4 (2.6–4.5)	**<0.001**

Parameters are described as number (n (%)), mean (standard deviation) or median (interquartile range) as appropriate. IgM-RF = immunoglobulin M rheumatoid factor; ACPA = anti-citrullinated protein antibodies; ESR = erythrocyte sedimentation rate; CRP = C-reactive protein; DAS28 = disease activity score in 28 joints. Significance levels were set to p<0. 0056 when corrected for multiplicity.

**Fig 1:**
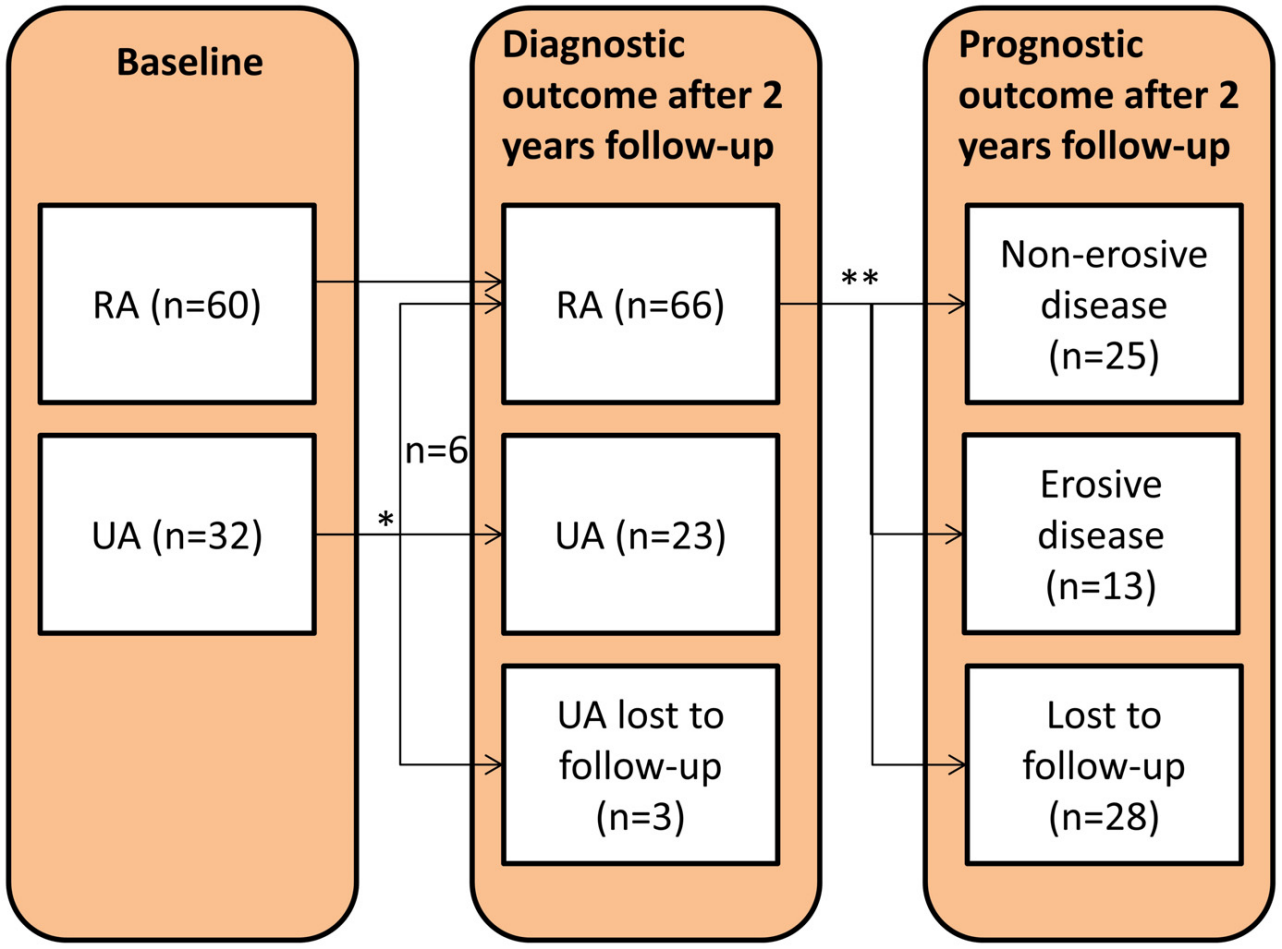
Schematic overview of the study. At baseline the selected patients either fulfilled the ACR/EULAR 2010 criteria for the classification of rheumatoid arthritis (RA; n = 60) or had unclassified arthritis (UA; n = 32). Patients were followed for 2 years and those with UA were categorized as having either converted to RA (UA-RA; n = 6) or remained unclassified (UA-UA; n = 23). Patients fulfilling the ACR/EULAR 2010 criteria for RA after the 2 years follow-up were further classified for arthritis outcome into non-erosive disease (n = 25) or erosive disease (n = 13). * Three patients were not available for follow-up and were therefore excluded from the diagnostic outcome analysis. ** Of 28 patients the arthritis outcome data were not available and were therefore excluded from the prognostic outcome analysis.

Age and gender were comparable between the RA and UA patients. IgM-RF positivity and ACPA positivity were lower in the UA group compared to the RA group. Baseline ESR and CRP were comparable between the groups, whereas DAS28 and ACPA positivity were higher in the RA group. A schematic overview of the study is presented in Fig 1.

### Baseline biomarker concentrations are higher in RA patients compared to UA patients

We observed a significantly higher concentration of, C3M and CRPM in RA compared to UA based on the diagnosis at baseline (p<0.001 and p = 0.004, respectively). In patients diagnosed with RA, the median (IQR) baseline concentrations of C3M, and CRPM were 34.1 ng/mL (24.5–41.2) and 12.8 ng/mL (9.2–16.0), respectively. For UA diagnosis at baseline, the median (IQR) concentration of C3M and CRPM were, 23.7 ng/mL (21.5–29.4) and 9.6 ng/mL (7.6–12.4.), respectively. C1M had a tendency to be higher in patients diagnosed with RA with a median (IQR) level of 37.7ng/ml (26–64.1) compared to patients with UA with the median level of 27.7ng/ml (20.4–51.3) (p = 0.028). There was no difference in concentrations of C2M or VICM between the RA and UA diagnosis at baseline (p = 0.84 and p = 0.13, respectively).

The analysis was followed by the investigation of the biomarkers in relationship to diagnostic outcome. After 2 years follow-up period, 6 (18%) of the patients initially classified as UA fulfilled the classification criteria for RA. The patients were stratified in one of following three groups considering their baseline and 2 years follow-up diagnosis: UA-UA (n = 23), UA-RA (n = 6) (three UA patients were lost to follow-up) and RA-RA (n = 60). C3M levels was significantly different between the groups (p = 0.005). C3M concentrations (median (IQR)) were higher in RA-RA (34.1 ng/mL (24.6–41.2)) compared to UA-RA (23.9 ng/mL (20.40–31.9)) and UA-UA (24.1 ng/mL (21.6–29.6)). CRPM concentrations was also higher RA-RA (12.8 ng/mL (9.2–16.0)) than in UA-UA (9.6 ng/mL (7.6–12.0)) and UA-RA (10.8 ng/mL (7.2–14.8)). There was no statistically significant difference in concentrations of C1M, C2M and VICM between the different diagnostic outcome groups (p = 0.16, p = 0.90 and p = 0.23, respectively).

### Diagnostic power of biomarkers to discriminate between RA and UA diagnosis

To evaluate the diagnostic power of the biomarkers of joint destruction and inflammation and of other disease activity markers to discriminate between the UA and RA groups regarding baseline diagnosis, we calculated the area under the receiver operating characteristic (AUROC). The diagnostic power of C1M, C3M and CRPM was highly significant with an AUROC of 0.64 (95% CI 0.52 to 0.76), 0.73 (95% CI 0.62 to 0.84) and 0.68 (95% CI 0.57 to 0.80), respectively (Table 2). The same evaluation was performed with CCP and the disease activity parameter DAS28 resulted in AUROC values of 0.85 (95% CI 0.762 to 0.914) and 0.74 (95% CI 0.64 to 0.84) respectively. C2M, VICM, CRP and ESR were not sufficient to reach statistical significance.

**Table 2:** Area under the receiver operating characteristic (AUROC) for discriminating between RA and UA baseline diagnosis.

Biomarker	AUROC	Std. Error	95% confidence interval	P value
C1M	0.64	0.062	0.52 to 0.77	0.028
C2M	0.51	0.065	0.39 to 0.64	0.838
C3M	0.73	0.055	0.62 to 0.84	<0.001
VICM	0.60	0.061	0.48 to 0.72	0.130
CRPM	0.68	0.057	0.57 to 0.80	0.004
ESR	0.61	0.060	0.49 to 0.73	0.080
CRP	0.60	0.062	0.48 to 0.72	0.117
DAS28	0.74	0.052	0.64 to 0.84	<0.001
CCP	0.85	0.039	0.76 to 0.91	<0.001

Significance levels were set to p<0. 0056 when corrected for multiplicity.

An univariate logistic analysis was used to assess the relationship between C1M, C2M, C3M, VICM, CRPM, ESR, CRP, DAS28, RF positivity and ACPA positivity, on one hand, and baseline diagnosis on the other. C3M was related to baseline diagnosis (OR 1.07, 95% CI 1.02 to 1.13, p = 0.006), and there was an association between CRPM and baseline diagnosis (OR 1.78, 95% CI 1.14 to 2.77, p = 0.012). Evaluated clinical parameters were significantly associated with baseline diagnosis: DAS28 (OR 1.94, 95% CI 1.34 to 2.80, p<0.001), RF positivity (OR 43.4, 95% CI 5.6 to 340, p<0.001), and ACPA positivity (OR 26.6, 95% CI 7.1 to 99.4, p<0.001).

Next, we performed multivariate logistic regression analysis with C3M and CRPM in combination with RF positivity, ACPA positivity and/or DAS28. C3M and CRPM did not reach statistical significance in this analysis.

We also calculated the AUROC to assess the ability of the biomarkers to discriminate between patients who subsequently progressed from UA to RA after 2 years and patients who remained UA after 2 years. None of the soluble biomarkers could statistically significantly predict whether patients would be in the UA-RA group compared to the UA-UA group (Table 3).

**Table 3:** Area under the receiver operating characteristic (AUROC) for discriminating between UA patients that progress to RA after 2 years of follow-up and those that remain UA after 2 years of follow-up.

Biomarker	AUROC	Std. Error	95% confidence interval	P value
C1M	0.49	0.12	0.24 to 0.73	0.91
C2M	0.52	0.13	0.26 to 0.79	0.87
C3M	0.49	0.13	0.23 to 0.76	0.96
VICM	0.32	0.12	0.09 to 0.55	0.18
CRPM	0.45	0.14	0.17 to 0.73	0.71
ESR	0.68	0.14	0.41 to 0.94	0.19
CRP	0.61	0.15	0.32 to 0.90	0.42
DAS 28	0.37	0.17	0.04 to 0.70	0.33
CCP	0.53	0.14	0.34 to 0.72	0.82

Significance levels were set to p<0. 0056 when corrected for multiplicity.

### Diagnostic power of biomarkers to discriminate between *non-erosive* and *erosive disease* after 2 years follow-up

Prognostic outcome after 2 years follow-up was assessed in 66 patients with RA. 28 (42%) patients could not be classified due to missing data. There were no significant differences in baseline characteristics (including age, DAS28, sex or CRP) between the remaining patients and those that were lost to follow-up. Among the remaining RA patients two years after initiation of the study, 25 (38%) had *non-erosive disease* and 13 (20%) had *erosive disease*; Baseline C2M concentrations were slightly higher in patients with *erosive* disease (0.23 ng/mL (0.19–0.26)) compared to patients with non-erosive disease (0.18 ng/mL (0.15–0.22) as a prognostic outcome (p = 0.011), this difference was borderline significant when adjusted for multiple testing, with the highest (median (IQR)) concentration in the *erosive* group and the lowest concentration in the *non-erosive* group. Baseline C1M, C3M, VICM and CRPM were comparable between the prognostic outcome groups.

This analysis was followed by the investigation of the power of the biomarkers to discriminate between groups with different prognostic outcome. The prognostic power of C2M had an AUROC of 0.75 (95% CI 0.60 to 0.91) (Table 4). Baseline C1M, C3M, VICM, CRPM or any of the standard clinical parameters ESR, CRP, CCP, and DAS28 were not statistically significant predictors of outcome in this study.

**Table 4:** Area under the receiver operating characteristic (AUROC).

Biomarker	AUROC	Std. Error	95% confidence interval	P value
C1M	0.51	0.11	0.3 to 0.71	0.96
C2M	0.75	0.08	0.60 to 0.91	0.01
C3M	0.51	0.11	0.30 to 0.73	0.89
VICM	0.57	0.10	0.38 to 0.76	0.50
CRPM	0.57	0.11	0.36 to 0.79	0.47
ESR	0.51	0.10	0.31 to 0.71	0.93
CRP	0.59	0.10	0.40 to 0.78	0.38
DAS28	0.46	0.10	0.27 to 0.66	0.70
CCP	0.62	0.09	0.49–0.73	0.18

AUROCs discriminating between *non-erosive* and *erosive disease* for patients fulfilling the RA criteria after 2 years of follow-up. Significance levels were set to p<0. 0056 when corrected for multiplicity.

Next, univariate logistic analysis was applied to assess the relationship between C1M, C2M, C3M, VICM, CRPM, ESR, CRP, DAS28, RF positivity and ACPA positivity on the one hand and prognostic outcome on the other. None of the markers reached statistical significance using this approach.

### Biomarker concentrations correlate significantly with clinical disease activity measurements

The correlations between the biomarkers of joint destruction and inflammation and measures of clinical disease activity were assessed in the overall population and separately (Table 5) in the patients diagnosed with RA at baseline (Table 6). All soluble biomarkers tested showed statistically significant correlations with measures of disease activity.

**Table 5:** Correlations between baseline biomarker concentration and other parameters for disease activity in the overall population (UA+RA).

	DAS28	ESR	CRP	SJC66	TJC68	HAQ
*C1M p*	**<0.001**	**<0.001**	**<0.001**	**0.003**	0.040	**0.004**
*r*	**0.53**	**0.66**	**0.87**	**0.31**	0.21	**0.30**
*C2M p*	0.15	0.029	0.067	0.22	0.93	0.34
*r*	0.15	0.23	0.19	0.13	-0.010	-0.10
*C3M p*	**<0.001**	**<0.001**	**<0.001**	**0.001**	**0.009**	0.14
*r*	**0.63**	**0.73**	**0.65**	**0.34**	**0.27**	0.16
*VICM p*	0.019	0.13	0.015	0.039	0.047	0.11
*r*	0.25	0.16	0.25	0.22	0.21	0.17
*CRPM p*	**<0.001**	**<0.001**	**<0.001**	**0.003**	0.10	0.12
*r*	**0.47**	**0.60**	**0.60**	**0.31**	0.17	0.28

The correlations between the biomarkers of joint destruction and inflammation and measures of clinical disease activity were assessed in the overall population (UA+RA) at baseline. UA = unclassified arthritis; RA = rheumatoid arthritis; DAS28 = disease activity score in 28 joints; ESR = erythrocyte sedimentation rate; CRP = C-reactive protein; SJC66 = 66 swollen joint score; TJC68 = 68 tender joint score; HAQ = health assessment questionnaire; C1M = matrix metalloproteinases (MMP) degraded type I collagen; C2M = MMP degraded type II collagen; C3M = MMP degraded type III collagen; VICM = citrullinated and degraded vimentin; CRPM = MMP degraded CRP. Significance levels were set to p<0.01 when corrected for multiplicity.

**Table 6:** Correlations between baseline biomarker concentration and other parameters for disease activity in the population with RA.

	DAS28	ESR	CRP	SJC66	TJC68	HAQ
*C1M p*	**<0.001**	**<0.001**	**<0.001**	**0.007**	0.16	**0.003;**
*r*	**0.57**	**0.69**	**0.89**	**0.35**	0.18	**0.38**
*C2M p*	0.024	0.026	0.079	0.19	0.45	0.88
*r*	0.29	0.29	0.23	0.19	0.10	0.02
*C3M p*	**<0.001**	**<0.001**	**<0.001**	**0.004**	0.12	0.15
*r*	**0.60**	**0.69**	**0.66**	**0.37**	0.20	0.19
*VICM p*	0.15	0.35	0.27	0.37	0.59	0.15
*r*	0.19	0.12	0.15	0.12	0.07	0.19
*CRPM p*	**<0.001**	**<0.001**	**<0.001**	0.010	0.28	0.13
*r*	**0.48**	**0.58**	**0.58**	0.33	0.14	0.20

The correlations between the biomarkers of joint destruction and inflammation and measures of clinical disease activity in the patients diagnosed with RA at baseline. RA = rheumatoid arthritis; DAS28 = disease activity score in 28 joints; ESR = erythrocyte sedimentation rate; CRP = C-reactive protein; SJC66 = 66 swollen joint score; TJC68 = 68 tender joint score; HAQ = health assessment questionnaire; C1M = matrix metalloproteinases (MMP) degraded type I collagen; C2M = MMP degraded type II collagen; C3M = MMP degraded type III collagen; VICM = citrullinated and degraded vimentin; CRPM = MMP degraded CRP. Significance levels were set to p<0. 01 when corrected for multiplicity.

### C1M and C3M can discriminate between patients in remission and those with persistent disease activity

Next, we investigated the power of the biomarkers to discriminate between patients in remission (DAS<2.6) versus those with active disease. First, we evaluated the power of C1M and C3M to discriminate between remission versus active disease in UA and RA patients (n = 10 patients in remission; n = 82 patients with active disease) and found that these were significant with an AUROC of 0.75 (95% CI 0.62 to 0.88) and 0.79 (95% CI 0.68 to 0.89), respectively. C2M, VICM, CRPM did not appear to provide any information in this regard. Secondly, we performed this analysis in RA patients only (n = 3 patients in remission; n = 57 patients with active disease) and found that C1M, C3M and CRPM were significant with an AUROC of 0.84 (95% CI 0.68 to 1.00), 0.94 (95% CI 0.87 to 1.00), and 0.91 (95% CI 0.80 to 1.00), respectively.

## Discussion

This early arthritis study investigated five neo-epitopes (C1M, C2M, C3M, VICM, CRPM), which are soluble biomarkers each representing different aspects of joint destruction and inflammation. We aimed to determine the ability of these biomarkers to improve the current diagnostic and/or prognostic process in early arthritis patients and to investigate the tissue turnover in the joints reflected by these biomarkers in early arthritis patients. The applied biomarkers evaluate neo-epitopes released during tissue turnover, and they are therefore sensitive measures of alterations during pathological events in inflamed tissue. We investigated the biomarker profile in early RA as well as in early UA patients. We demonstrated that early RA is associated with significantly increased serum levels of C3M and CRPM hence increased connective tissue turnover compared to UA. Also C1M levels appeared to be increased in RA compared to UA although this tendency was not significant after Bonferroni correction for multiple testing. Furthermore, our results indicates that turnover of cartilage, reflected by C2M, levels was elevated in early RA patients who subsequently developed erosive disease after 2 years of follow-up.

The diagnostic utility of the biomarkers was explored in early RA patients and we found that the biomarkers C1M, C3M and CRPM all had a diagnostic power which was comparable to the standard disease parameter DAS28 when evaluated as AUROC. This is not unexpected since RA is characterized by massive changes of metabolic processes in the joints, which includes cartilage degradation and connective tissue turnover as a consequence of synovial inflammation. Thus C1M, C3M and CRPM add further information to the well-established clinical parameters. However the diagnostic power of CCP was exceeding all of the evaluated biomarkers as well as DAS28 with an AUROC value of 0.85. All of the applied biomarkers correlated with one or more of the established parameters for disease activity, such as DAS28, ESR, and/or CRP. These results presented here support the notion that connective tissue degradation relates to the inflammatory process in RA. However, the soluble biomarkers tested were unable to predict which of the UA patients at baseline would eventually fulfil classification criteria for RA since the levels of soluble biomarkers were not statistically significantly different between the UA-RA group and the UA-UA group. Single use of a biomarker may therefore not provide enough diagnostic information since RA is a very heterogeneous disease.

Type I, II and III collagen are the main collagens in the joint. Therefore, an increase in MMP fragments of these collagens may provide novel information about connective tissue balance. Indeed, earlier studies demonstrated an association between elevated levels of serum C2M and severity of osteoarthritis, suggesting that C2M could be applied as a biomarker for cartilage loss or degradation [27]. This is in line with our study, which indicated that C2M concentrations were different between the prognostic outcome groups, with a tendency of higher concentrations in *erosive* disease.

These findings suggest that the biomarkers may contribute with independent and additive information about the disease pathogenesis and may provide supplementary diagnostic tools for clinical diagnosis. These biomarkers should not compete with current diagnostic tools for clinical diagnosis or disease activity. Instead they provide additional information on tissue integrity.

A limitation of this study is the relatively small sample size; there was a limited number of UA-RA patients (n = 6). Larger studies including higher numbers of early arthritis patients followed longitudinally are needed to confirm these initial findings. In addition it would be preferable to include healthy and age matched non-arthritic patients for the comparison.

This study shows for the first time that measurement of C1M, C3M, and CRPM may assist in differential diagnosis in early arthritis patients. In addition, C2M might be a prognostic biomarker predictive of the development of erosive disease. The results provide the rationale for larger studies in early arthritis patients to confirm and extend these findings.

Raw data of demographics and clinical and laboratory parameters as well as biomarker measurements underlying the presented findings are available in the S1 Dataset.

## Supporting Information

S1 Dataset (XLS)
